# Reliable ECG Anomaly Detection on Edge Devices for Internet of Medical Things Applications

**DOI:** 10.3390/s25082496

**Published:** 2025-04-15

**Authors:** Moez Hizem, Leila Bousbia, Yassmine Ben Dhiab, Mohamed Ould-Elhassen Aoueileyine, Ridha Bouallegue

**Affiliations:** 1 Innov’COM Laboratory, Higher School of Communication of Tunis, University of Carthage, Tunis 1054, Tunisia; leila.bousbiaa@gmail.com (L.B.); yassmine.bendhiab@supcom.tn (Y.B.D.); ridha.bouallegue@supcom.tn (R.B.); 2 Faculty of Sciences of Tunis, University of Tunis El Manar, Tunis 2092, Tunisia

**Keywords:** TinyML, edge AI, IoMT, anomaly detection, ECG, low-power, embedded systems

## Abstract

The advent of Tiny Machine Learning (TinyML) has unlocked the potential to deploy machine learning models on resource-constrained edge devices, revolutionizing real-time monitoring in Internet of Medical Things (IoMT) applications. This study introduces a novel approach to real-time electrocardiogram (ECG) anomaly detection by integrating TinyML with edge Artificial Intelligence (AI) on low-power embedded systems. We demonstrate the feasibility and effectiveness of deploying optimized models on edge devices, such as the Raspberry Pi and Arduino, to detect ECG anomalies, including arrhythmias. The proposed workflow encompasses data preprocessing, feature extraction, and model inference, all executed directly on the edge device, eliminating the need for cloud resources. To address the constraints of memory and power consumption in wearable devices, we applied advanced optimization techniques, including model pruning and quantization, achieving an optimal balance between accuracy and resource utilization. The optimized model achieved an accuracy of 92.3% while reducing the power consumption to 0.024 mW, enabling continuous, long-term health monitoring with minimal energy requirements. This work highlights the potential of TinyML to advance edge AI for real-time medical applications.

## 1. Introduction

The proliferation of Internet of Things (IoT) devices and the emergence of Tiny Machine Learning (TinyML) have revolutionized healthcare applications, enabling the deployment of machine learning models on resource-constrained devices [1,2,3]. Among these, real-time health monitoring has gained significant attention, particularly for cardiovascular health, where continuous monitoring of electrical heartbeat signals (using ECGs) is critical for the early detection and management of conditions such as arrhythmias [4,5]. The traditional ECG monitoring systems often rely on centralized data processing, which presents challenges such as high latency, elevated power consumption, and privacy concerns [6]. These limitations make centralized systems suboptimal for wearable real-time health monitoring devices.

Edge AI [7], powered by TinyML, provides a transformative solution by enabling real-time data processing directly on low-power embedded systems [2]. This approach reduces the dependency on cloud computing, thereby the minimizing latency and power usage while improving data privacy. Recent advances in microcontrollers, equipped with sensors and Bluetooth Low Energy (BLE) modules, have made wearable health monitoring applications feasible [8]. Their low power consumption and sufficient computational capabilities allow for the implementation of optimized TinyML models for real-time anomaly detection. Leveraging these capabilities, this study aims to develop a robust, real-time ECG anomaly detection system that continuously monitors heart activity and promptly alerts users to potential health issues [4].

Cardiovascular diseases remain a leading cause of morbidity and mortality worldwide, highlighting the need for efficient and reliable monitoring solutions [5]. ECG signals are a vital source of information about heart health, with anomalies indicating conditions such as atrial fibrillation, bradycardia, and other arrhythmias [9]. Early detection of these anomalies is crucial to preventing severe complications and improving patient outcomes. However, the traditional ECG systems, which depend on bulky equipment and centralized analyses, are impractical for continuous real-time monitoring in everyday settings.

**Figure 1:**
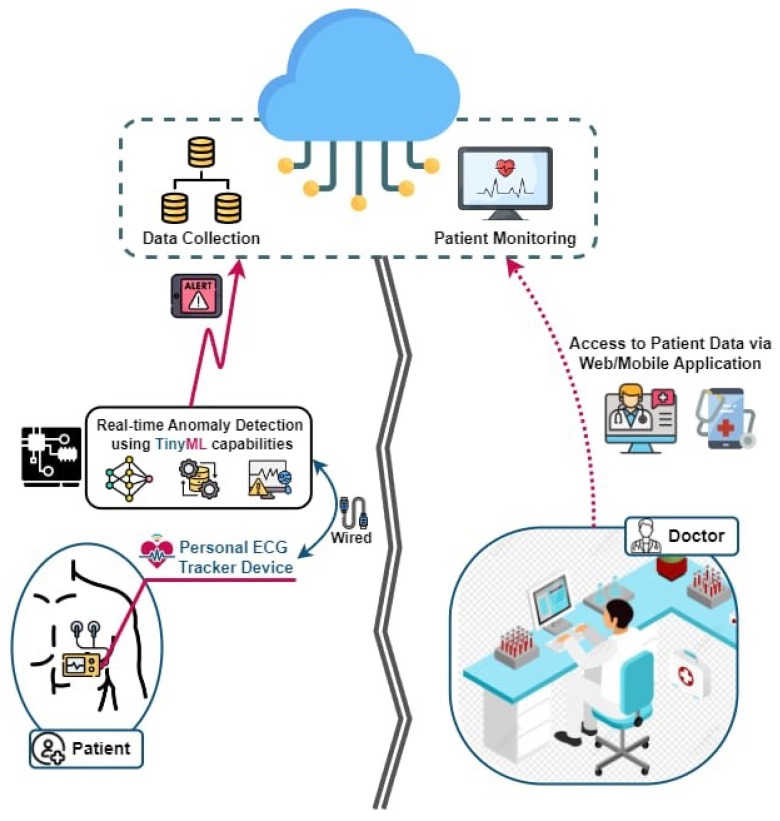
Real-time edge anomaly detection using TinyML platform.

By integrating TinyML with IoT-enabled devices, the proposed system enables a continuous, real-time analysis of ECG signals directly on wearable devices [10], as given in Figure 1, which illustrates the architecture of the proposed system. This integration eliminates the need for constant data transmission to central servers, conserving bandwidth, reducing the power consumption, and ensuring the privacy of sensitive health data. Furthermore, the miniaturization of sensors and advancements in microcontroller technologies have enabled the design of compact, low-power devices capable of running sophisticated machine learning algorithms.

In this study, we propose a convolutional neural network (CNN)-based model for ECG anomaly detection. CNNs are particularly effective for analyzing time-series data, as they can automatically learn and extract relevant features from raw data. We trained the model using publicly available ECG datasets, such as the MIT-BIH Arrhythmia database [11], which offers a diverse range of annotated ECG signals. The trained model was then optimized for deployment on the Raspberry Pi using TensorFlow Lite, a lightweight version of TensorFlow designed for mobile and embedded systems.

To ensure efficiency and reliability, the proposed methodology includes several critical steps: data preprocessing to improve the signal quality, model optimization to reduce the size and enhance the inference speed, and a rigorous evaluation of the performance in terms of accuracy, latency, and power consumption. Quantization techniques were also applied to minimize the model’s footprint without compromising its predictive accuracy.

The performance of the proposed system was evaluated through extensive experiments that calculated its precision in detecting ECG anomalies, the latency of real-time inference, and the overall power consumption during continuous operation. The results demonstrate that the optimized system achieves high accuracy in anomaly detection while maintaining low power consumption, making it well suited to continuous monitoring in wearable devices. The system also ensures low latency, enabling timely alerts for detected anomalies.

## 2. Understanding ECG Signals

Electrocardiographs (ECGs) [12] are a widely used, non-invasive technique for monitoring the heart’s electrical activity over time. An ECG’s waveform consists of distinct components, with each corresponding to specific physiological events in the cardiac cycle. A thorough understanding of these components and intervals is essential to assessing cardiac function and identifying potential abnormalities. The intervals in the ECG waveform provide an overarching view of the cardiac cycle:

- The PR interval: This reflects the time taken for electrical signals to travel from the atria to the ventricles. It includes the P wave and the PR segment, representing atrial depolarization and the delay at the atrioventricular (AV) node, respectively.
- The QT interval: This represents the total duration of ventricular activity, encompassing both depolarization and repolarization. Abnormalities in the QT interval are often linked to arrhythmias and other cardiac conditions.

**Figure 2:**
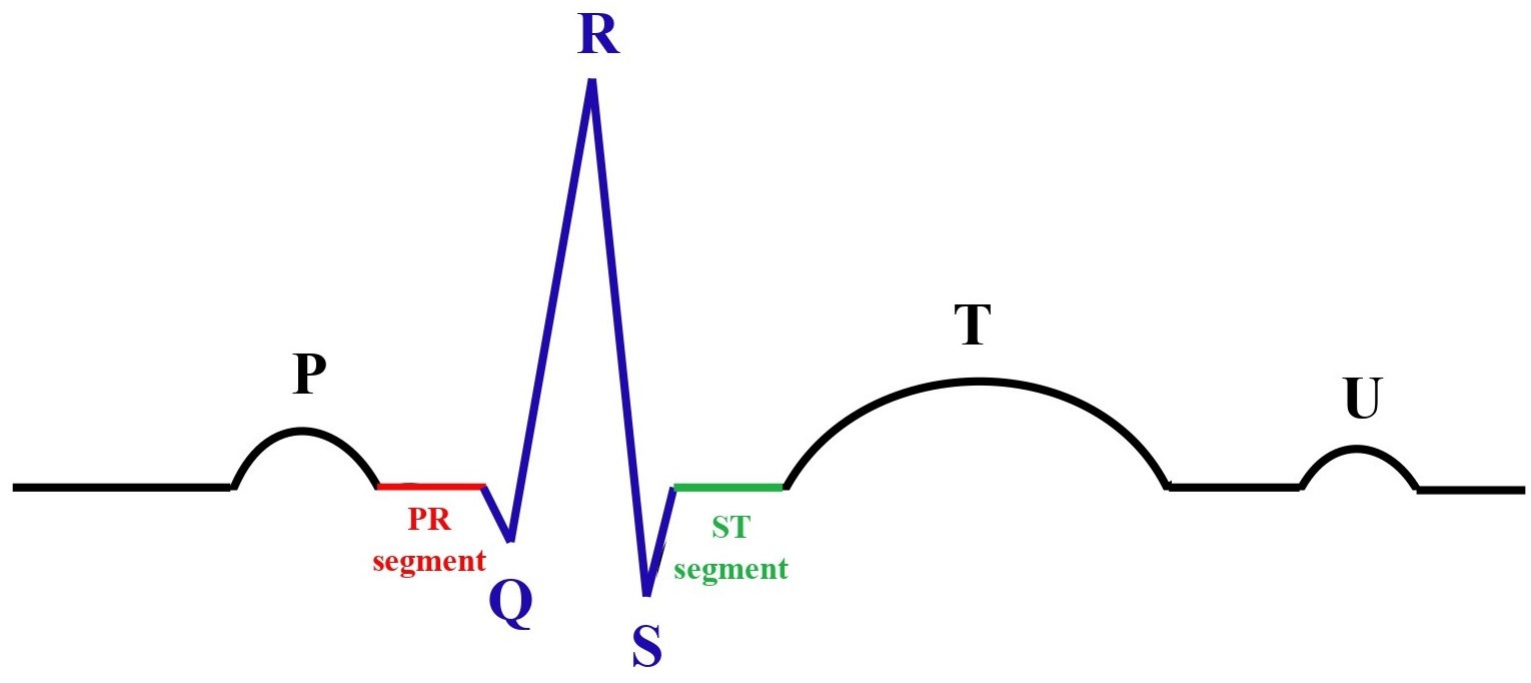
ECG signal details.

Following these intervals, the key waveform components, as shown in Figure 2, include the following:

1. The P wave: This reflects the electrical depolarization of the atria, which initiates atrial contraction and marks the start of the cardiac cycle.
2. The PR segment: This corresponds to the period of the electrical conduction delay at the AV node, allowing sufficient time for the ventricles to fill with blood before contraction.
3. The QRS complex: This represents the rapid depolarization of the ventricles, triggering ventricular contraction. This is the most prominent feature of an ECG and a key marker for cardiac rhythm.
4. The ST segment: This denotes the interval between ventricular depolarization and repolarization. Under normal conditions, this segment appears relatively flat, with deviations often indicative of ischemic or other pathological changes.
5. The T wave: This reflects ventricular repolarization, which restores the ventricles to their resting state and prepares them for the next contraction cycle.
6. The U wave: Occasionally observed after the T wave, the U wave’s origin remains uncertain but is thought to be related to late repolarization phenomena, such as repolarization of the Purkinje fibers.

A detailed and systematic examination of these intervals and waveform components enables clinicians to evaluate cardiac function comprehensively and diagnose a variety of conditions, including arrhythmias, ischemia, and conduction disorders. Such an analysis is fundamental to guiding clinical decisions and therapeutic interventions.

## 3. Anomaly Detection in ECG Signals

ECG signals serve as a vital diagnostic tool, providing a comprehensive view of the heart’s electrical activity. These signals play a crucial role in identifying and diagnosing a range of cardiovascular conditions. Anomalies in ECG signals, such as arrhythmias or ischemic changes, often signify serious cardiac issues, including irregular heart rhythms, myocardial infarction, or conduction abnormalities. The ability to detect such anomalies in real time is particularly important in applications such as wearable health monitoring systems, where timely medical intervention can significantly improve patient outcomes [9].

### 3.1. Types of ECG Anomalies

ECG anomalies can manifest in various forms, reflecting underlying cardiac dysfunctions. Some of the most common types include the following:

- Arrhythmia: This refers to irregularities in heart rhythm. These may present as the following:
  – Tachycardia: A rapid heart rate exceeding 100 beats per minute;
  – Bradycardia: A slow heart rate below 60 beats per minute;
  – Fibrillation (AF): An irregular and often rapid rhythm arising from disorganized atrial activity.
- Premature ventricular contractions (PVCs): These are characterized by early contractions of the ventricles, which may feel like a “skipped” heartbeat. These are often benign but may indicate more severe underlying conditions if frequent.
- ST-segment elevation: This is an indicative marker of acute myocardial infarction (a heart attack). It is characterized by an abnormal upward shift in the ST segment of the ECG waveform, signaling injury to the myocardium.

Each type of anomaly provides unique insights into cardiac health and guides clinicians in customizing the treatment strategies.

### 3.2. Anomaly Detection Using Edge AI

Detecting ECG anomalies in real time is a complex yet essential task, particularly in wearable and portable healthcare systems. This study employs TinyML models deployed on edge devices, such as the Raspberry Pi and Arduino, to achieve efficient and timely anomaly detection. The process involves preprocessing the raw ECG signals, followed by feature extraction and anomaly classification.

The key features analyzed by the model include heart rate variability (HRV), QRS complex duration, and other temporal and spectral features derived from the ECG waveform. These features enable the model to classify the ECG signals as normal or abnormal, identifying specific anomalies such as arrhythmias or ischemic changes.

The deployment of anomaly detection systems on edge devices offers significant advantages over cloud-based solutions. By eliminating the need for continuous data transmission to remote servers, these systems reduce latency, enhance data privacy, and improve response times. Additionally, the lightweight nature of TinyML models ensures compatibility with resource-constrained environments, making them ideal for real-time monitoring applications.

### 3.3. Model Optimization and Accuracy

To ensure that the anomaly detection system meets the constraints of edge devices, this study incorporates advanced model optimization techniques. Methods such as pruning and quantization were employed to significantly reduce the model’s size and computational requirements while preserving its predictive accuracy. These optimizations make the model not only feasible for real-time deployment but also energy-efficient, which is critical for wearable and portable health monitoring systems.

Experimental evaluations demonstrated that the optimized model achieved a high classification accuracy across various types of ECG anomalies, underscoring its potential for widespread clinical and personal health applications. By balancing accuracy with computational efficiency, the proposed system addresses the dual challenges of scalability and real-time responsiveness in resource-limited settings.

## 4. Related Works

Recent advancements in TinyML have shown promising results in deploying machine learning models on embedded systems for various applications, including health monitoring [13]. Studies have demonstrated the effectiveness of edge AI in reducing the latency and power consumption while maintaining high accuracy [14]. However, there is a need for further exploration into optimizing these models for specific health applications, such as real-time ECG anomaly detection, which requires high sensitivity and specificity. The intersection of Tiny Machine Learning, the Internet of Things, and e-health has seen significant research interest, particularly in the realm of real-time health monitoring. Several studies have explored the deployment of machine learning models on resource-constrained devices for various health applications, with a focus on ECG anomaly detection being one of the critical areas.

One important study in this domain was conducted by A. Ashfaq et al. (2022), where the authors of [15] developed a deep learning model for arrhythmia detection using ECG signals. The model, based on a convolutional neural network (CNN), achieved high accuracy but was designed for execution on powerful servers rather than on edge devices. This study highlighted the potential of deep learning for ECG analysis but also underscored the need to optimize the model for edge deployment.

Building on this, Negin Alamatsaz et al. (2024) [16] demonstrated the application of deep learning in ECG analysis through a CNN-based approach that could identify a wide range of arrhythmias. While their model achieved impressive results, its deployment was still limited to environments with substantial computational resources, pointing to a gap in research focused on low-power, edge-based solutions.

The concept of TinyML was explored extensively by E. A. Essanoah Arthur et al. (2024) [17], who discussed the challenges and opportunities in deploying machine learning models on microcontrollers and other low-power devices. Their work emphasized the importance of model compression techniques, such as quantization and pruning, to enable the execution of sophisticated models on resource-constrained hardware. This paper provided valuable insights into the technical considerations necessary for implementing TinyML in real-world applications, including health monitoring.

The study in [18] aimed to design a TinyML workflow, outlining the steps for model creation and deployment in resource-limited environments, and then implemented this workflow in e-health applications for real-time epileptic seizure detection using electroencephalography (EEG) data.

Specific to ECG monitoring, D. Amiri et al. (2022) [19] proposed an edge computing framework for wearable ECG monitoring devices. Their system employed a lightweight deep learning model for arrhythmia detection, optimized for deployment on an edge device. They reported significant reductions in the latency and power consumption compared to these properties in traditional cloud-based solutions. This study demonstrated the feasibility of an edge-based ECG analysis but suggested further optimization to enhance the model’s efficiency.

In terms of practical implementations, Alimbayeva, Z. et al. (2024) [20] presented a wearable ECG monitoring system using a low-power microcontroller. Their system incorporated a CNN model for real-time arrhythmia detection, demonstrating the viability of deploying TinyML models on wearable devices. They focused on optimizing both the hardware and software aspects to achieve low power consumption, making their system suitable for continuous monitoring applications.

The work by Tahir, Sidra et al. (2024) [21] explored the use of federated learning to train ECG anomaly detection models across multiple devices without sharing the raw data. This approach enhanced data privacy and enabled collaborative model improvements while keeping the data decentralized. Although their study primarily focused on the training phase, it opened avenues for deploying these collaboratively trained models on edge devices for real-time inference.

Furthermore, Nong, X. et al. (2023) [22] investigated the integration of multimodal sensor data to improve the accuracy of health monitoring systems. They combined ECG data with other physiological signals to enhance the robustness of the anomaly detection models. Their findings suggested that multimodal approaches could significantly improve the detection accuracy but also highlighted the challenges in processing and integrating diverse data streams on low-power devices.

Lastly, the study by G. Cerutti et al. (2020) [23] on optimizing neural networks for energy-efficient inference on microcontrollers provided valuable guidelines for developing low-power health monitoring solutions. Their techniques for model compression and energy-aware scheduling were crucial in making real-time ECG monitoring feasible on wearable devices.

Table 1 provides a comparison between some of the references mentioned above. We also added other studies which handled the same context as that of our study [24,25,26,27]. This is in order to illustrate some details about the literature.

These studies collectively underscore the progress and ongoing challenges in the deployment of TinyML for the real-time detection of ECG anomalies. Although significant advances have been made in optimizing the machine learning models for edge devices, there remains a continuous need for innovative approaches to improve the accuracy, efficiency, and power consumption in real-world health monitoring applications.

## 5. The Proposed Work

This section details the methodology employed to develop a robust and optimized ECG anomaly detection system for deployment on resource-constrained embedded devices. The proposed workflow integrates data processing, machine learning model development, optimization techniques, and deployment strategies to achieve real-time performance and energy efficiency. The key stages include the following:

1. Data processing and preparation: This involves utilizing the MIT-BIH Arrhythmia dataset, addressing class imbalances using advanced oversampling techniques such as SMOTE, and extracting meaningful features for training the model.
2. Machine learning model development: Four distinct models (a CNN, decision tree, Random Forest, and XGBoost) were trained and evaluated to determine the most suitable algorithm for ECG anomaly detection. The CNN model was selected for its superior performance in terms of the accuracy and F1-score.
3. Model optimization for edge AI and TinyML: To ensure efficient deployment on embedded devices, pruning and quantization techniques were applied, significantly reducing model size and power consumption without compromising performance.
4. Deployment on embedded devices: The optimized model was deployed on the Raspberry Pi 4 and Arduino Nano using an AD8232 ECG sensor. The hardware setup was designed to enable real-time inference, with live monitoring of the ECG signals and anomaly detection.
5. Performance evaluation and comparative analysis: The optimized model’s performance was evaluated in terms of accuracy, latency, throughput, and energy efficiency, both on host and target devices. A comparative analysis with existing works highlights the advantages of the proposed approach.

By following this comprehensive workflow, the proposed system demonstrates the feasibility of deploying machine learning models for real-time ECG anomaly detection in low-power, embedded environments. Each stage of the methodology is detailed in the subsequent sections.

### 5.1. Data Processing and Preparation

#### 5.1.1. Overview of the Dataset

**Figure 3:**
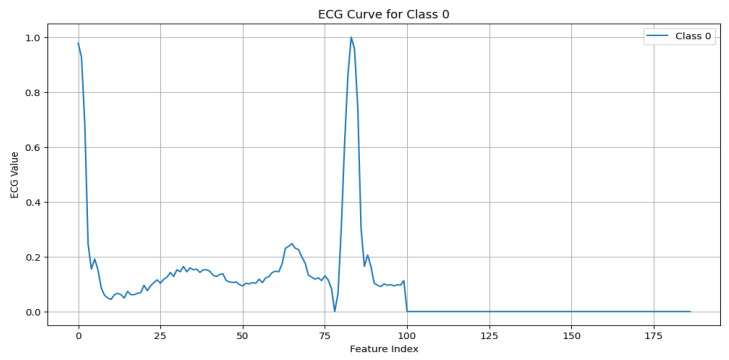
ECG signal for normal beat.

**Figure 4:**
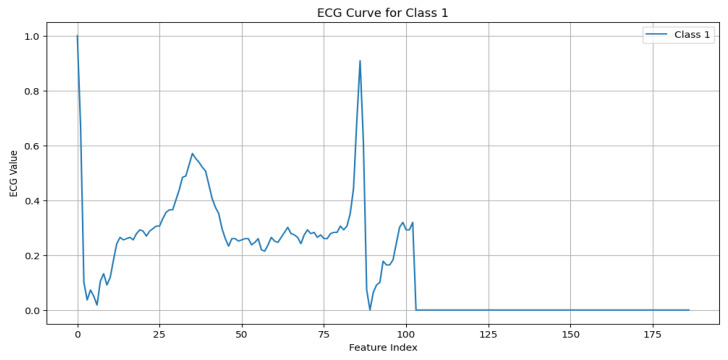
ECG signal for supraventricular premature beat.

**Figure 5:**
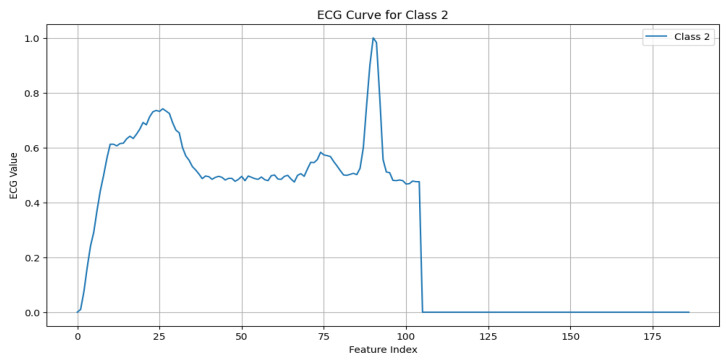
ECG signal for premature ventricular contraction.

**Figure 6:**
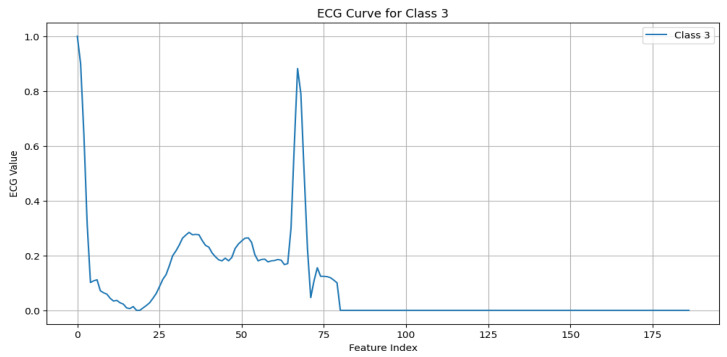
ECG signal for fusion of ventricular and normal beats.

**Figure 7:**
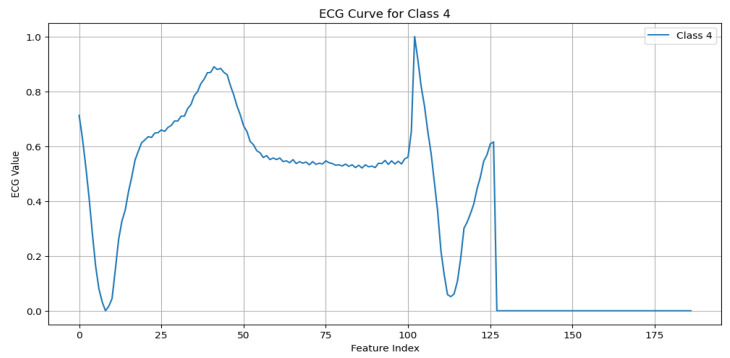
ECG signal for unclassifiable beat.

The MIT-BIH Arrhythmia dataset [11] serves as the foundational dataset for this work, offering a diverse set of annotated ECG signals critical for developing and validating ECG anomaly detection models. The dataset includes five primary beat classes, with each representing unique cardiac activity, as detailed below:

- N (Normal beat): This represents typical QRS complexes with no abnormalities, indicative of a “Normal Sinus Rhythm”. Key characteristics include regular rhythm, the consistent presence of the P wave, and normal PR intervals (120–200 ms) and QRS durations (80–120 ms), as shown in Figure 3.
- S (Supraventricular premature beat): This arises from ectopic atrial or AV node activity, resulting in early beats. It is characterized by possible abnormal or hidden P waves, as shown in Figure 4.
- V (Premature ventricular contraction): This represents early ventricular contractions with no preceding P wave, wide QRS complexes, and opposite-direction T waves, as shown in Figure 5.
- F (Fusion of ventricular and normal beats): This occurs when the supraventricular and ventricular beats coincide, creating a hybrid waveform, as shown in Figure 6.
- Q (Unclassifiable beat): This includes beats with irregular morphologies due to noise or artifacts, requiring further investigation, as shown in Figure 7.

#### 5.1.2. Data Preprocessing

**Figure 8:**
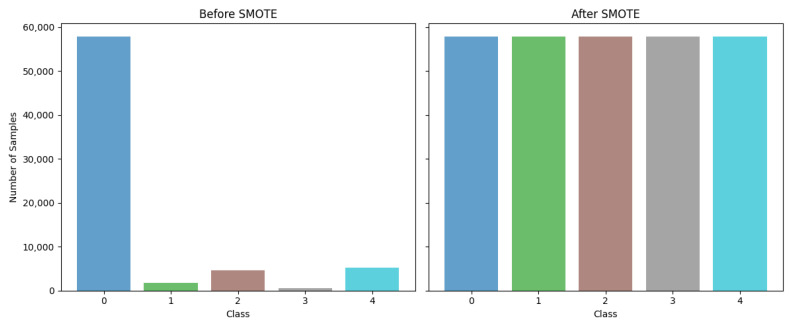
Data balancing using the SMOTE method.

To prepare the dataset for effective training, we followed a structured workflow, starting with addressing class imbalances using the Synthetic Minority Over-Sampling Technique (SMOTE) [28]. The SMOTE is a sophisticated resampling method that generates synthetic samples for minority classes by interpolating between the existing examples. Unlike simple duplication, this approach creates plausible, new instances that contribute to a balanced class distribution. Balancing the dataset is crucial to training robust and unbiased machine learning models, ensuring that minority classes, such as rare ECG anomalies, are accurately represented during training [29]. The improved balance reduces the risk of classifier bias towards the majority class, enhancing the performance of the model in minority predictions [30]. The balanced dataset yielded using the SMOTE is illustrated in Figure 8; as mentioned at the top of the figure, on the left are the original data and on the right the data balanced using SMOTE.

Following the dataset balancing, the ECG signals underwent comprehensive preprocessing to improve the signal quality and standardize the inputs for the model. The first step, **denoising**, involved applying a band-pass filter to remove noise and artifacts from the raw ECG signals, such as baseline wander and high-frequency interference. This ensured that only relevant components of the cardiac signal were retained [31]. Next, **normalization** was performed using min–max scaling, rescaling the signal amplitudes to a consistent range of [0, 1]. This step reduced the variability and ensured compatibility across samples [32]. Finally, the signals were **segmented** into overlapping 10-second windows with a stride of 1 s, isolating distinct cardiac patterns for model training and enabling accurate anomaly detection [33].

This integrated approach of balancing, denoising, normalization, and segmentation significantly enhanced the quality and usability of the dataset. By combining the SMOTE to address class imbalances with advanced preprocessing techniques, we ensured that the model could generalize well across all ECG beat types, maintaining fairness, accuracy, and robustness in predicting cardiac anomalies on resource-constrained embedded systems [34].

## 6. Machine Learning Model Development

The machine learning model development phase involved evaluating multiple algorithms to identify the most suitable model for ECG anomaly detection. The workflow included feature engineering, model selection, and a rigorous performance evaluation.

### 6.1. Feature Engineering

To extract meaningful insights from the preprocessed ECG data, feature engineering was conducted to transform the raw signals into representations that highlighted distinctive cardiac patterns. Dimensionality reduction techniques, such as a Principal Component Analysis (PCA), were used to simplify the input space while retaining essential information. Recursive Feature Elimination (RFE) was applied to prioritizing the most relevant features to the model training. These steps ensured that the models focused on high-impact attributes, improving their ability to differentiate between normal and abnormal ECG patterns.

### 6.2. Model Selection and Training

**Figure 9:**
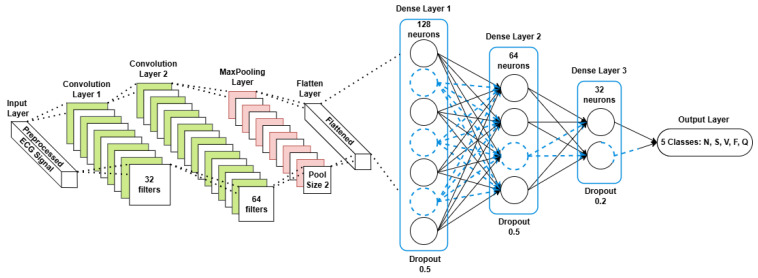
Proposed CNN model architecture.

Four machine learning algorithms were evaluated for their suitability for classifying ECG signals:

- Convolutional neural networks (CNNs): Chosen for their ability to automatically learn spatial hierarchies from the input data, CNNs performed exceptionally well in extracting features from the time-series ECG signals. The CNN workflow begins by preprocessing the raw 1D ECG signals and transforming them into a time–frequency representation using the Short-Time Fourier Transform (STFT). The resulting spectrograms serve as 2D inputs to the CNN, enabling it to extract both **temporal and spectral features through convolutional layers. This approach enhances feature learning, allowing the model to effectively classify ECG signals while optimizing the performance for deployment on embedded systems. Figure 9 illustrates the CNN workflow.
- Decision trees: These interpretable models split the dataset based on the feature importance, represented by a tree structure. Although simple and efficient, they are prone to overfitting [35].
- Random Forest (RF): This is an ensemble learning technique that aggregates multiple decision trees to enhance the accuracy and robustness. RF achieved a higher performance compared to that of standalone decision trees by reducing the overfitting [36].
- XGBoost: A gradient boosting algorithm known for its speed and regularization capabilities, which helps mitigate overfitting while maintaining high accuracy [37].

The models were trained using TensorFlow, with the dataset split into 80% for training and 20% for testing. Hyperparameter optimization, including learning rate and batch size tuning, was performed using a grid search to achieve the optimal performance.

To assess the model’s performance, the following metrics were computed [38]:

(1) $$\mathrm{Accuracy}=\frac{\mathrm{Number}\;\mathrm{of}\;\mathrm{Correct}\;\mathrm{Predictions}}{\mathrm{Total}\;\mathrm{Number}\;\mathrm{of}\;\mathrm{Predictions}}$$

(2) $$\mathrm{Precision}=\frac{\sum_{i=1}^{n}{\mathrm{TP}}_{i}}{\sum_{i=1}^{n}\left({\mathrm{TP}}_{i}+{\mathrm{FP}}_{i}\right)}$$

(3) $$\mathrm{Recall}=\frac{\sum_{i=1}^{n}{\mathrm{TP}}_{i}}{\sum_{i=1}^{n}\left({\mathrm{TP}}_{i}+{\mathrm{FN}}_{i}\right)}$$

(4) $$F1-\mathrm{Score}=\frac{2\times \mathrm{Precision}\times \mathrm{Recall}}{\mathrm{Precision}+\mathrm{Recall}}$$

Here, $TP_{i}$ represents the true positives for class *i*, $FP_{i}$ the false positives, $FN_{i}$ the false negatives, and *n* the total number of classes.

**Figure 10:**
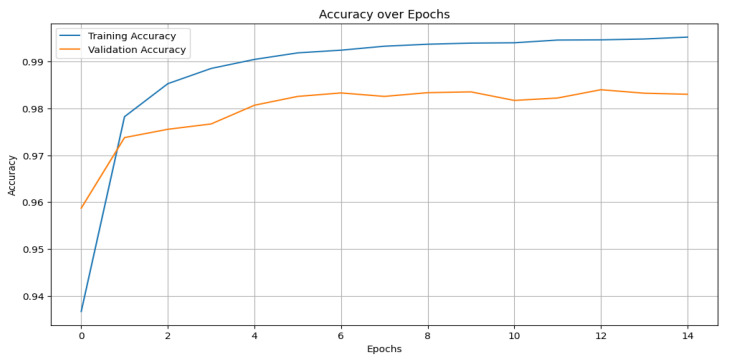
Training and validation curve for the best model (CNN).

**Figure 11:**
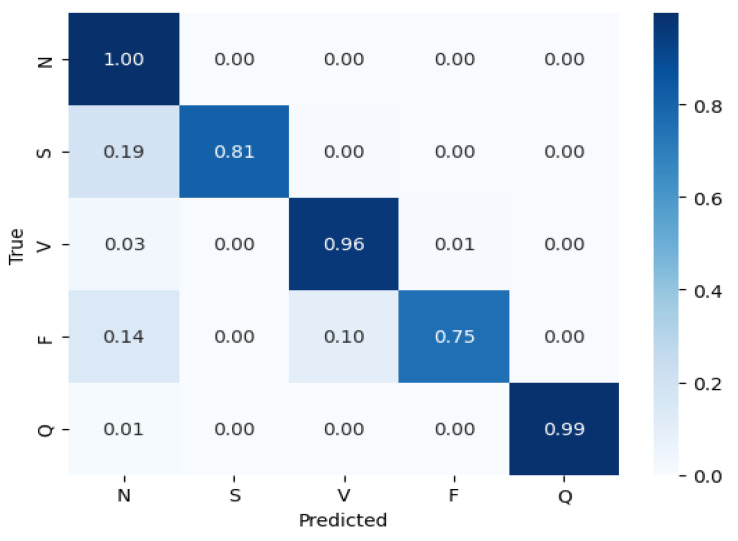
Confusion matrix for the best model (CNN).

Comparative evaluation: The performance of all of the models was assessed using these metrics. Table 2 presents the comparative results, where the CNN outperformed the other models with an accuracy of 99%, precision of 98%, recall of 98%, and an F1-score of 98%. Figure 10 shows the training and validation accuracy across epochs for the CNN model, while Figure 11 depicts its confusion matrix, highlighting its classification effectiveness.

### 6.3. Model Selection and Training

**Figure 12:**
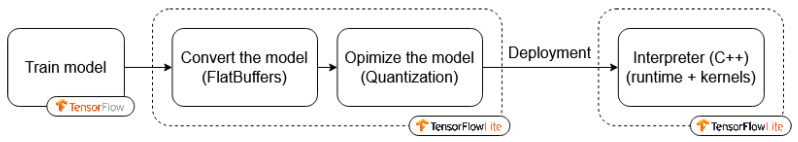
Workflow from ML to TinyML for the chosen models.

Four machine learning algorithms were evaluated for their suitability in classifying ECG signals:

- **Convolutional neural networks (CNNs)**: Chosen for their ability to automatically learn spatial hierarchies from the input data, CNNs have performed exceptionally well in extracting features from time-series ECG signals [34]. The architecture of the proposed CNN model is detailed in Figure 9. It consists of the following:
  – **Convolutional layers:** Extracted local features using a kernel size of 3;
  – **Pooling layers:** Applied max pooling to reduce the dimensionality while retaining critical features;
  – **Dropout layers:** Prevented overfitting by randomly deactivating a fraction of the neurons during training;
  – **Fully connected layers:** Mapped the extracted features to the output space for multi-class classification;
  – **Activation functions:** A Rectified Linear Unit (ReLU) was used for the hidden layers, while softmax was applied in the output layer.
  Figure 12 illustrates the overall CNN workflow, from training to deployment.
- **Decision trees:** These interpretable models split the dataset into subsets based on the feature importance, represented by a tree structure. Although simple and efficient, they are prone to overfitting [35].
- **Random Forest (RF):** An ensemble learning technique that aggregates multiple decision trees to enhance the accuracy and robustness. RF achieved a higher performance compared to that of the standalone decision trees by reducing the overfitting [36].
- **XGBoost:** A gradient boosting algorithm known for its speed and regularization capabilities, which helps mitigate overfitting while maintaining high accuracy [37].

The models were trained using TensorFlow, with the dataset split into 80% for training and 20% for testing. Hyperparameter optimization, including the learning rate and batch size tuning, was performed using a grid search to achieve the optimal performance. The CNN model, in particular, was trained with the following parameters:

- **Learning rate:** 0.001;
- **Optimizer:** The Adam optimizer;
- **Batch size:** 32;
- **Epochs:** 50.

### 6.4. The Evaluation Metrics

To assess the model’s performance, the following metrics were computed:

(5) $$\mathrm{Accuracy}=\frac{\mathrm{Number}\;\mathrm{of}\;\mathrm{Correct}\;\mathrm{Predictions}}{\mathrm{Total}\;\mathrm{Number}\;\mathrm{of}\;\mathrm{Predictions}}$$

(6) $$\mathrm{Precision}=\frac{\sum_{i=1}^{n}{\mathrm{TP}}_{i}}{\sum_{i=1}^{n}\left({\mathrm{TP}}_{i}+{\mathrm{FP}}_{i}\right)}$$

(7) $$\mathrm{Recall}=\frac{\sum_{i=1}^{n}{\mathrm{TP}}_{i}}{\sum_{i=1}^{n}\left({\mathrm{TP}}_{i}+{\mathrm{FN}}_{i}\right)}$$

(8) $$F1-\mathrm{Score}=\frac{2\times \mathrm{Precision}\times \mathrm{Recall}}{\mathrm{Precision}+\mathrm{Recall}}$$

Here, $TP_{i}$ represents the true positives for class *i*, $FP_{i}$ the false positives, $FN_{i}$ the false negatives, and *n* the total number of classes.

### 6.5. Comparative Evaluation

The performance of all of the models was assessed using these metrics. Table 2 presents the comparative results, where the CNN outperformed the other models with an accuracy of 99%, precision of 98%, recall of 98%, and an F1-score of 98%. The effectiveness of the CNN model is further demonstrated in Figure 10, which shows the training and validation accuracy across epochs, and Figure 11, which depicts its confusion matrix.

## 7. Model Optimization for Edge AI and TinyML

To ensure its efficient deployment on resource-constrained devices, the selected CNN model was optimized using pruning and quantization. These techniques significantly reduced the model’s size and computational requirements while maintaining a high performance for real-time ECG anomaly detection.

### 7.1. Model Pruning

Pruning is a model optimization technique that removes redundant or less significant weights and neurons from a neural network, reducing its size and computational complexity [39]. In this work, magnitude-based pruning was applied post-training. Weights with the smallest magnitudes were iteratively pruned, balancing size reduction and accuracy retention.

### 7.2. Model Quantization

Quantization reduces the precision of the model weights and activations from 32-bit floating-point to 8-bit integers, drastically lowering the memory usage and computational demands [40]. Post-training quantization was applied using TensorFlow Lite, enabling efficient deployment on devices with limited computational power.

### 7.3. Combined Optimization: Pruning and Quantization

To achieve the best balance of accuracy and lightweightness, pruning and quantization were combined. The pruning step reduced the model size, while quantization enhanced the inference speed and memory efficiency. This combined approach ensured that the optimized model remained competitive in its accuracy while achieving significant reductions in its size and power consumption.

### 7.4. The Model Deployment Workflow

The optimization process was integrated into a TinyML workflow using TensorFlow Lite. Figure 12 illustrates the steps from model training to deployment, including optimization and conversion into a lightweight format compatible with embedded devices including the Raspberry Pi, ensuring adaptability and portability for real-time applications.

### 7.5. The Performance Results

Table 3 and Table 4 present the evaluation metrics for the original, pruned, quantized, and optimized models. The optimized model demonstrated substantial reductions in its size and computational demand while retaining a competitive accuracy, making it highly suitable for resource-constrained environments.

Power consumption is a critical factor in deploying AI models on edge devices, particularly in battery-operated and resource-constrained environments. In this study, the power consumption is estimated based on the CPU utilization during inference. The relationship between CPU usage and power consumption is approximated using a scaling factor of $P_{\mathrm{base}}=1.2$ mW per second of CPU utilization [41,42], derived from the typical power characteristics of ARM-based processors. The power consumption for each model is estimated using Equation (9). Furthermore, the energy per inference is calculated using Equation (10).

(9) $$\mathrm{Power}\;\mathrm{Consumption}\;\left(\mathrm{mW}\right)=P_{\mathrm{base}}\times \mathrm{Average}\;\mathrm{Inference}\;\mathrm{Time}\;\left(s\right)$$

where $P_{base}$ is the base power consumption in mW.

(10) EnergyperInference(J)=PowerConsumption(mW)×AverageInferenceTime(s)

where the operations per layer are the product of the dimensions of the weights and activations for that layer; Equation (11) gives the expression of the FLOPs.

(11) $$\mathrm{FLOPs}=\sum_{\mathrm{layers}}\left(\mathrm{Operations}\;\mathrm{per}\;\mathrm{Layer}\right)$$

As shown in Table 4, this metric provides insight into the efficiency of each model in terms of the energy consumption per prediction, which is crucial for real-time, continuous monitoring applications in e-health and IoT-based smart healthcare systems. The estimated and calculated results demonstrate that pruning and quantization significantly reduce the power consumption, making the optimized models more suitable for edge deployment. Future work will involve power measurements using hardware-based energy profiling tools to refine these estimations further.

## 8. Prototype of the Target Device and Its Deployment

The model’s performance was evaluated in terms of accuracy, latency, and power consumption. Comparisons were made with baseline models running on more powerful hardware to demonstrate the feasibility of our approach. The final prototype was deployed and tested using the following components:

- A Raspberry Pi 4 [43];
- An Arduino Nano [44];
- An AD8232 ECG sensor [45];
- A screen display for real-time monitoring.

Table 5 gives the details of the embedded devices used.

**Figure 13:**
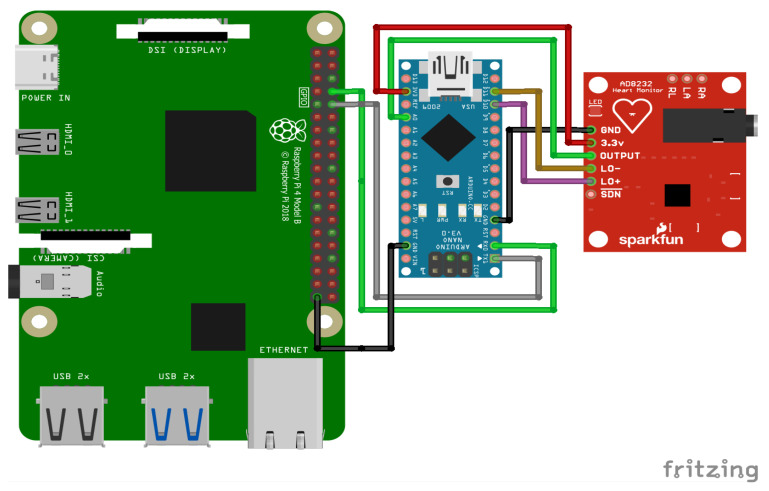
Wiring and deployment schematic for the edge device, showing connections between the Raspberry Pi, the Arduino Nano, and the AD8232 ECG sensor.

An overview of the final electronic wiring of the prototype is provided in Figure 13, including the connections between the Raspberry Pi, the Arduino Nano, and the AD8232 ECG sensor. The experimental setup consists of an Arduino Nano interfaced with an AD8232 ECG sensor to acquire real-time electrocardiogram (ECG) signals. Electrodes are placed on the patient’s body to record real-time signals. The acquired analog signals are digitized and transmitted to the Raspberry Pi via a Universal Asynchronous Receiver–Transmitter (UART) serial communication link with a baud rate of 9600 bps. Given that each transmitted data point consists of a single byte (8 bits), along with the start and stop bits, the effective transmission time per byte is approximately 1.04 ms. This delay accumulates over continuous data transmission, introducing a latency factor that affects real-time processing. The Raspberry Pi, acting as the edge computing device, receives the ECG data, processes them, and performs inference using a pre-trained TinyML model. To mitigate the impact of the serial link delay, an optimized data transmission protocol is employed, ensuring efficient buffering and synchronization between the Arduino and the Raspberry Pi.

**Figure 14:**
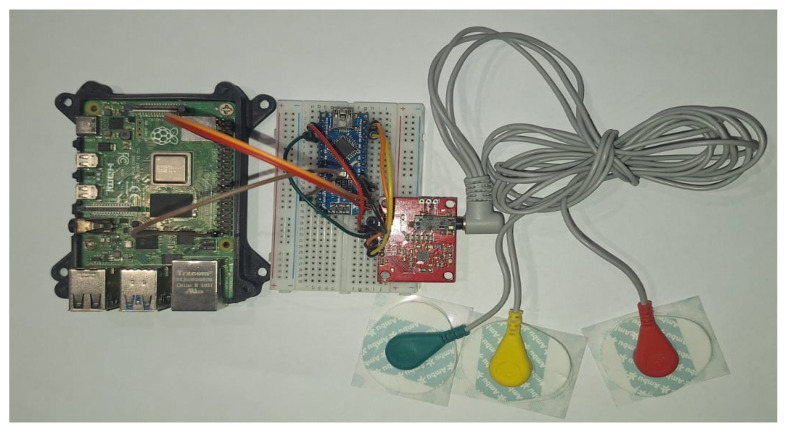
Real device for ECG data collection using the AD8232 ECG sensor, the Arduino Nano, and the Raspberry PI.

To collect ECG data, the AD8232 sensor is connected to the Arduino Nano, as shown in Figure 14.

**Figure 15:**
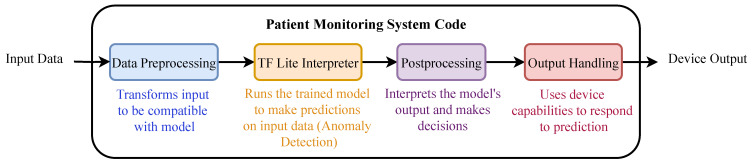
Workflow of the deployed algorithm, detailing the data collection, preprocessing, and inference steps.

The ECG signals are acquired through the AD8232 sensor, preprocessed by the Arduino Nano, and sent to the Raspberry Pi for inference. Real-time monitoring and anomaly detection results are displayed on a connected screen. The workflow of the deployed model is illustrated in Figure 15.

## 9. Results and Discussion

### 9.1. The Performance Evaluation Metrics

The performance of the optimized models was evaluated using key metrics that quantified their suitability for edge deployment. These included the following:

(12) $$\mathrm{Model}\;\mathrm{Size}\;\left(\mathrm{KB}\right)=\frac{\mathrm{File}\;\mathrm{Size}\;\left(\mathrm{Bytes}\right)}{1024}$$

(13) $$\mathrm{Compression}\;\mathrm{Ratio}=\frac{\mathrm{Original}\;\mathrm{Model}\;\mathrm{Size}\;\left(\mathrm{KB}\right)}{\mathrm{Compressed}\;\mathrm{Model}\;\mathrm{Size}\;\left(\mathrm{KB}\right)}$$

(14) $$\mathrm{Average}\;\mathrm{Inference}\;\mathrm{Time}=\frac{\sum_{i=1}^{n}t_{i}}{n}$$

(15) $$\mathrm{Throughput}=\frac{1}{\mathrm{Average}\;\mathrm{Inference}\;\mathrm{Time}}$$

Additional metrics, such as the power consumption, CPU usage, and memory utilization, were calculated to assess the model’s operational efficiency. These metrics provide valuable insights into the trade-offs between the accuracy, energy efficiency, and computational requirements, as summarized in Table 3 and Table 4.

### 9.2. Comparison and Evaluation of Our Proposed Models

The comparative performance of the proposed models for the detection of ECG anomalies on resource-limited embedded devices, such as the Raspberry Pi, is summarized in Table 6 and Table 7. The “original” model achieved the highest accuracy of 96.09% but exhibited a significantly higher power consumption (0.4 µW) and inference time (0.333 ms), making it less practical for continuous monitoring in resource-constrained environments.

**Figure 16:**
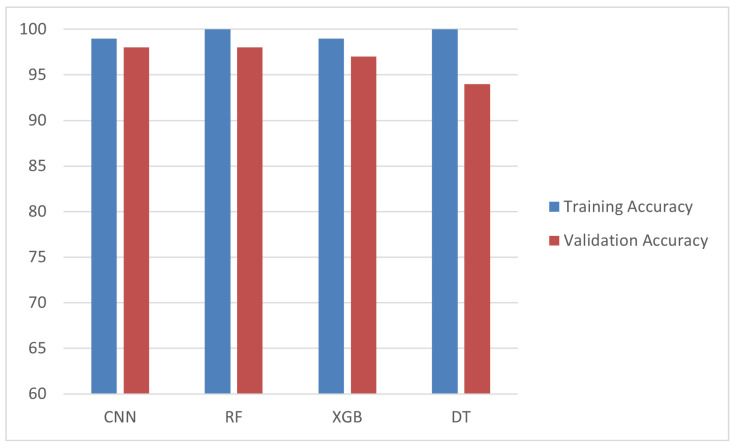
Comparison of accuracy across models.

By applying pruning, the power consumption of the model was kept to 0.4 µW while maintaining an acceptable accuracy of 92 89%. Quantization alone provided a balance between the performance and resource efficiency, achieving a precision of 96.01% and drastically reducing the power consumption to 0.4 µW. The “optimized” model, which combined both pruning and quantization, achieved the lowest power consumption of 0.24 µW while maintaining a competitive accuracy of 92 89%. Figure 16 illustrates the accuracy of these models, highlighting the suitability of the ”optimized” model for real-time health monitoring on embedded devices.

Additional metrics, including the inference time, throughput, and resource usage, are detailed in Table 7. The “optimized” model achieved the fastest inference time of 0.200 ms, a throughput of 5008.04 inferences per second, and the lowest CPU usage of 15.4%. This shows its efficiency for real-time health monitoring applications. Furthermore, the quantized model achieved the highest throughput of 5204.03 inferences per second and the lowest latency values, making it a suitable choice for scenarios where both high performance and low power consumption are critical.

### 9.3. Comparison with the Existing Approaches

The performance of the proposed model was compared with existing works in ECG anomaly detection, as summarized in Table 8. While leveraging the same dataset (MIT-BIH Arrhythmia) across the evaluations, our model demonstrates significant advantages in its accuracy, lightweightness, and edge suitability. For instance, the Spiking Neural Network (SNN) with a CAM by [46] achieved a high accuracy of 98.2% but relied on FPGA systems, which lack the accessibility and flexibility of platforms like the Raspberry Pi. In contrast, our optimized model achieved an accuracy of 99.0%, with a significantly reduced power consumption and inference time, making it ideal for real-time, resource-constrained environments.

### 9.4. Discussion

The results highlight the effectiveness of leveraging TinyML techniques, such as pruning and quantization, to optimize ECG anomaly detection models for resource-constrained devices. As shown in Table 6 and Table 7, the “optimized” model balanced its accuracy (92.89%) with a significantly reduced power consumption (0.24 µW) and inference time (0.200 ms), demonstrating its suitability for real-time IoT healthcare applications. Compared with existing works, the model outperformed them in its accuracy (99.0%) and demonstrated a superior edge suitability, bridging the gap between computational efficiency and accuracy for low-power systems.

However, this study has limitations, including the use of a single dataset (MIT-BIH Arrhythmia) and deployment on a single platform (the Raspberry Pi). Future work will address these limitations by evaluating the model on diverse datasets and alternative embedded devices, such as the Arduino or ESP32. Additionally, integrating multimodal data (e.g., ECGs and EEG) and employing advanced techniques such as neural architecture search (NAS) could further enhance the framework’s applicability and performance.

## 10. Conclusions and Future Works

In this paper, we presented a novel approach to ECG anomaly detection using a CNN-based model optimized through pruning and quantization for deployment on low-power embedded systems like the Raspberry Pi. Our optimized model achieved an exceptional performance on the target device, with an accuracy of 99%, precision of 98%, recall of 98%, and F1-score of 98%, demonstrating its suitability for real-time, resource-constrained environments. By leveraging Tiny AI techniques, we showcased that accurate and efficient anomaly detection can be achieved on edge devices, offering a practical solution for continuous health monitoring in remote or home-based care settings.

This work distinguishes itself by successfully combining a high model accuracy with optimizations tailored to low-power hardware, addressing the dual challenges of computational efficiency and real-time processing. Unlike previous studies, which often prioritized either accuracy or edge device compatibility, our approach bridges this gap, underlining the transformative potential of Tiny AI in making advanced healthcare solutions accessible through portable and affordable devices.

Looking ahead, there are several avenues for further improvements and expansion. Firstly, integrating advanced model compression techniques, such as knowledge distillation or neural architecture search (NAS), could minimize the model’s size and computational overhead further while maintaining or even enhancing the accuracy. Secondly, extending this framework to addressing other health-related conditions, such as detecting anomalies related to Parkinson’s disease or Alzheimer’s, would broaden its scope and applicability in the healthcare domain. Additionally, deploying this system on wearable devices for continuous, real-time processing could revolutionize personal health monitoring and pave the way for next-generation IoT healthcare systems.

Future work could also focus on improving the interpretability of the model by incorporating explainable AI (XAI) techniques. This would provide valuable insights into the model’s decision-making process, making it more transparent and acceptable in clinical settings. Furthermore, extending the framework to support multimodal data (e.g., combining ECG and EEG signals) would enhance its diagnostic capabilities, enabling real-time monitoring and management of multiple health conditions. Lastly, rigorous validation of the model in real-world scenarios, including noisy environments and diverse patient populations, would further establish its robustness and reliability for widespread clinical adoption.

In conclusion, this study represents a significant step forward in enabling energy-efficient, real-time ECG anomaly detection on resource-constrained devices. By addressing key challenges in IoT healthcare systems, our approach lays a strong foundation for future advancements in portable and accessible health monitoring technologies.

## Figures and Tables

**Table 1:** Literature study and comparison.

Title	Year	Main Focus	Data Types Used	Methodologies	Evaluation Metrics
[15]	2022	Arrythmia detection	MIT-BIH	Hybrid CNN-LSTM	recall, precision, F1, accuracy
[16]	2024	Arrythmia detection	MIT-BIH	CNN + LSTM	accuracy, sensitivity, specificity
[17]	2024	TinyML for maize leaf disease	Mendeley Data, Harvard Database	CNN	memory consumption, complexity, power consumption, accuracy, optimization
[19]	2022	SIC-EDGE ECG compression for wearable systems	MIT-BIH, MIT-SVDB	CNN	F1, recall, ROC
[20]	2024	Heart monitoring	Wearable ECG device	LR, DT, RF, SVM, XG-Boost, CNN	accuracy, F1
[22]	2023	Structural health monitoring	IPG-SHM-2020	DNN, MC-CNN	F1, precision, recall
[24]	2024	Evaluation of both gel and embodied electrodes	Clinical notes	Regression model	MAE, MSE, R2
[25]	2023	Heart disease classification	PhysioNet	NB, DT, SVM, ENS	F1, accuracy
[26]	2023	ECG user authentication	PTB database	CNN, LSTM	accuracy, recall, precision, F1, AUC
[27]	2023	Cardiac arrhythmia detection	PhysioNet	ResNet 50, AlexNet	accuracy, recall, precision, sensitivity, F1, AUC

**Table 2:** Algorithm performance comparison.

	Accuracy (%)	Precision (%)	Recall (%)	F1-Score (%)
CNN	99	98	98	98
Decision Tree	78	73	76	93
RF	98	97	98	98
XGBoost	97	97	97	97

**Table 3:** Key performance metrics on a host device for the optimized models.

Model	Accuracy (%)	Precision (%)	Recall (%)	F1-Score (%)	Model Size (KB)
Original	96.09	96.76	96.09	96.33	739.945
Pruned	92.90	95.86	92.90	93.97	739.945
Quantized	96.01	96.70	96.01	96.26	192.914
Optimized	92.89	95.83	92.89	93.96	193.359

**Table 4:** Extended comparison on a host device of the optimized models.

Model	Comp Ratio	Avg Inference Time (ms)	Throughput (Inferences/s)	Worst-Case Latency (ms)	95th-Percentile Latency (ms)	99th-Percentile Latency (ms)	FLOPs	Power (μW)	Energy (mJ)
Original	1.351	0.104	9583.166	14	0.138	0.185	226,832.0	52	5.408
Pruned	1.351	0.078	12,786.941	2.5	0.127	0.154	226,832.0	39	3.042
Quantized	5.184	0.045	22,114.766	2.1	0.073	0.107	226,832.0	23	1.035
Optimized	5.172	0.063	15,849.495	3.4	0.108	0.137	235,664.0	32	2.016

**Table 5:** Comparison of Raspberry Pi 4 and NodeMCU’s characteristics.

Characteristics	Raspberry Pi 4	NodeMCU (ESP8266)
**Processor**	Quad-core Cortex-A72 (ARM v8)	ESP8266 (32-bit Tensilica L106)
**Clock Speed**	1.5 GHz	80 MHz (can be overclocked to 160 MHz)
**Memory (RAM)**	2 GB, 4 GB, or 8 GB LPDDR4	32 KB instruction, 80 KB data RAM
**Storage**	MicroSD slot (up to 32 GB)	Flash memory: 4 MB (external up to 16 MB)
**Wi-Fi**	802.11 ac (dual-band 2.4/5 GHz)	802.11 b/g/n (2.4 GHz)
**Bluetooth**	Bluetooth 5.0, BLE	None
**GPIO Pins**	40 pins	10 pins
**USB Ports**	2x USB 3.0, 2x USB 2.0	None
**Ethernet**	Gigabit Ethernet (1000 Mbps)	None
**Power Consumption**	3.4 W (idle)–7.6 W (max)	170 mA (max)
**Operating Voltage**	5 V	3.3 V
**Power Supply**	USB-C 5 V/3 A	Micro-USB 5 V
**Dimensions**	85.6 mm × 56.5 mm	48 mm × 25 mm
**Weight**	46 g	7 g
**Operating System**	Raspberry Pi OS (Linux-based)	None (custom firmware like the Arduino, Lua, or MicroPython)

**Table 6:** Key performance metrics on target device (Raspberry Pi 04) of optimized models.

Model	Accuracy (%)	Precision (%)	Recall (%)	F1-Score (%)
Original	96.09	96.76	96.09	96.33
Pruned	92.89	95.86	92.89	93.97
Quantized	96.01	96.70	96.01	96.26
Optimized	92.89	95.83	92.89	93.96

**Table 7:** Detailed performance metrics on target device (Raspberry Pi 04) of optimized models.

Model	Avg Inference Time (ms)	Throughput (inferences/s)	Worst-Case Latency (ms)	95th-Percentile Latency (ms)	99th-Percentile Latency (ms)	Avg CPU Usage (%)	Power Consumption (μW)
Original	0.333	3004.76	9.638	0.379	0.397	72.4	0.4
Pruned	0.333	3004.89	0.791	0.377	0.396	47.5	0.4
Quantized	0.192	5204.03	0.347	0.211	0.246	31.5	0.23
Optimized	0.200	5008.04	0.426	0.226	0.252	15.4	0.24

**Table 8:** Comparison of models for ECG anomaly detection.

Research Paper	Model	Dataset	Number of Samples	Application	Accuracy (%)	Precision (%)	Recall (%)	F1-Score (%)	Platform	Edge Suitability
**Our Work**	CNN with Pruning + Quantization	MIT-BIH Arrhythmia	109446 (train + test)	ECG Anomaly Prediction	99.0	98.0	98.0	98.0	Raspberry Pi	Yes
[46]	Spiking Neural Network (SNN) + CAM	MIT-BIH Arrhythmia	109446 (train + test)	ECG Classification for Real-Time Implementation	98.2	93.66	94.75	90.9	FPGA	Partial
[47]	Hybrid Neural Network (HNN)	MIT-BIH Arrhythmia	109446 (train + test)	ECG Signal Classification with FIR Filter	92.3	93.4	98.5	90.6	GPU	No
[48]	ANN to SNN with LIF Neuron	MIT-BIH Arrhythmia	101200 (train + test)	ECG Classification	93.8	94.3	94.0	95.5	Host Device (CPU)	Partial

## Data Availability

The research data supporting this publication are available from the corresponding author upon reasonable request.
